# Secular Trends of the Impact of Overweight and Obesity on Hypertension in Yi People: Yi Migrant Study, 1996–2015

**DOI:** 10.1155/2020/5368357

**Published:** 2020-03-29

**Authors:** Jia Zhang, Shaoping Wan, Fen Dong, Li Pan, Wuli Yihuo, Haiying Gong, Fang Yang, Zheng Li, Guoju Li, Xiaoyang Wang, Guangliang Shan

**Affiliations:** ^1^Department of Epidemiology and Statistics, Institute of Basic Medical Sciences, Chinese Academy of Medical Sciences, School of Basic Medicine, Peking Union Medical College, Beijing 100005, China; ^2^Sichuan Cancer Hospital and Institute, Sichuan Cancer Center, Cancer Hospital Affiliated to School of Medicine, UESTC, Chengdu, Sichuan 610041, China; ^3^China-Japan Friendship Hospital, Beijing 100029, China; ^4^Puge County Center for Disease Control and Prevention, Liangshan, Sichuan 615300, China; ^5^Beijing Fangshan Center for Disease Control and Prevention, Beijing 102440, China; ^6^Xichang Municipal Center for Disease Control and Prevention, Xichang, Sichuan 615000, China; ^7^Qingdao Women and Children's Hospital, Qingdao University, Qingdao, Shandong 266011, China; ^8^The People's Hospital of Bao'an, Shenzhen, Guangdong 518101, China

## Abstract

**Background:**

Rising hypertension prevalence, coupled with increasing overweight and obesity rates, has been observed in Yi people. Moreover, the growing blood pressure level among Yi people was mostly attributable to the continuous increase of body mass index (BMI). However, little is known about the trend of association between them.

**Methods:**

Consequently, we investigated the impact of overweight/obesity on hypertension over three periods (1996, 2007-2008, 2015) using data from Yi Migrant Study (*n* = 8749). The Yi Migrant Study incorporated three successive cross-sectional studies which were implemented by the same team with consistent protocols.

**Results:**

Compared with period 1 (1996), the influence of overweight/obesity on hypertension risk significantly increased in period 2 (2007-2008) and period 3 (2015); relative excess risk due to interaction (RERI) was 1.59 (95% CI: 0.12, 3.05) and 1.41 (95% CI: 0.30, 2.78), respectively. Meanwhile, the overweight/obese population in period 3 did not show hypertension risk higher than that in period 2 (RERI = 0.15; 95% CI: −0.76, 1.07). Additionally, we observed a continuously growing trend of hypertension risk among normal weight Yi people.

**Conclusions:**

During the past two decades, there was a significant increase in the association between overweight/obesity and hypertension in Yi people, whereas the increasing trend has leveled off in more recent years. These findings suggest that overweight/obesity and hypertension are becoming more epidemic comorbidity over time. Interventions to prevent hypertension should focus not only on the overweight/obese population, but also on those with normal weight.

## 1. Introduction

Hypertension, also known as raised blood pressure (BP), is one of the most common medical disorders and the strongest modifiable risk factors of cardiovascular disease, which is a leading cause of death [1, 2]. During the past several decades, we witnessed an enormously increasing trend of hypertension prevalence worldwide [3–5]. Globally, one in four men and one in five women, in total 1.13 billion adults, had high blood pressure in 2015 [4]. Similarly, overweight/obesity is becoming a globally epidemic issue [6, 7]. As one of the most common risk factors for hypertension, excess weight gain can be the cause of 78% of primary hypertension in men and 65% in women [8]. The percentage of overweight/obesity among hypertension patients increased from 64.1% to 72.5% in US adults between 1999 and 2012 [9]. Moreover, 70.3% of overweight/obese individuals had hypertension, whereas the prevalence was only 15.3% in the non-overweight group [10].

The changing relationship between obesity and health outcomes is currently the subject of spirited debate [11]. While obesity is associated with increased risk for various adverse outcomes, there are signs that the effect of overweight/obesity on hypertension is dynamic. Among Japanese population, the impact of overweight and obesity on hypertension had a significant increase both in men and women during the past three decades [12]; for example, the odds ratio of overweight/obesity for hypertension increased from 1.94 in 1980 to 2.82 in 2010. Moreover, a comparison of the 1946 and 1958 British birth cohorts showed that the association between BMI trajectory and BP became stronger in the later-born cohort [13]. These unstable trends raise the question of whether overweight/obese persons in the most recent years are more likely to be hypertensive.

Yi people were characterized by low prevalence of hypertension and overweight/obesity. They were one of the least developed minority groups in China, which lead an extremely low socioeconomic status. In our previous studies, we found a significant increasing trend of prevalence both in hypertension and in overweight/obesity among Yi people during the past twenty years [14–16]. We also illustrated that the rapidly growing prevalence of hypertension in Yi people was primarily due to the parallel increasing mean value of BMI [14]. However, we did not quantify the trend in the relationship between hypertension and overweight/obesity, which could provide us with more insightful information on the public health level. Furthermore, as Yi people had been undergoing tremendous rural-urban migration since the 50s in last century, Yi migrants and Yi farmers are two distinct groups in terms of environmental factors but shared a common genetic background. It is undeniably useful for precision intervention and policy-making to elucidate the inequality between Yi farmers and Yi migrants. Consequently, in this study, our main purpose is to quantitatively explore potential trends of the relationship between overweight/obesity and hypertension among Yi people.

## 2. Materials and Methods

### 2.1. Study Design and Participants

The Yi Migrant Study includes three regionally representative cross-sectional studies (1996, 2007-2008, and 2015) in Liangshan Yi Autonomous Prefecture. The sampling procedures have been published previously in detail [14, 16, 17]. In the two earlier periods, stratified cluster sampling was used to select participants from Xichang city and Butuo, Zhaojue, Jinyang, Puge, and Xide counties. Due to limited data accessibility and study feasibility, we conducted investigation in Xichang city and Puge county in the last period.

Yi people, residing in Liangshan Yi Autonomous Prefecture located in Sichuan province, Southwestern China, used to be renowned for their extremely low hypertension prevalence and unchanged mean BP along with age [18]. The Yi farmers were defined as those whose parents were Yi people and have lived in the countryside since birth. Yi migrants were identified as those who were born in the countryside and then migrated to county or city for more than 1 year or those who were born in the urban area and have lived there until the survey. All Yi migrants' parents were certified Yi people.

The Yi ethnic group or Yi people is one of the biggest of China's 55 official minorities. There are about 9.5 million members in China. They live in southwestern China, both in mountains and in river valleys. The Liangshan Yi Autonomous Prefecture in southern Sichuan province has the largest density of Yi people. About 2 million Yi people live there, and this prefecture has almost all the Yi people in Sichuan. The Yi people usually live in remote mountain districts at or 1500 m above sea level. Their main occupation is agriculture, and they constitute one of the most primitive societies in China. The Yi farmers' settlements are isolated from the outside world, which means that only extremely steep and narrow paths could lead us to these villages. Additionally, they also preserved their own language, and most Yi farmers lack the ability to speak Mandarin Chinese. They make terraced fields and cultivate corn, buckwheat, and potatoes as staple food. Sour and spicy are their favourite tastes. They live in a relatively backward lifestyle, whereas the Yi migrants who live in the county or city with Han people have a much more modern lifestyle. It is comparatively convenient for them to acquire diverse food. And there was a considerable improvement in their living conditions compared with Yi farmers who started to migrate to the urban area from remote mountain districts since the 1950s [19].

### 2.2. Measurements

Data were collected by trained medical staff using standardized methods and identical examination protocol during the three time periods, including a questionnaire for demographic characteristics and several anthropometric measurements. In order to reduce information bias, we conducted a two-stage questionnaire survey. First, participants completed the questionnaire with trained medical staff. Then, several group leaders audited each questionnaire by randomly extracting some specific items.

All study proposals were approved by the review board of Institute of Basic Medical Sciences, Chinese Academy of Medical Sciences, School of Basic Medicine, Peking Union Medical College. Participant consent was required before the survey in the form of signature or fingerprint.

After an overnight fast, BP was measured by trained physicians using Mercury sphygmomanometer in 1996 and Omron automatic digital BP measuring device in 2007-2008 and 2015. Appropriate BP cuff sizes were used for participants based on measurement of the midarm circumference. BP was measured in the sitting position and on the right arm after a rest of at least 10 min; participants had not smoked, exercised, or eaten. Both systolic BP (SBP) and diastolic BP (DBP) were recorded. The mean of three measurements was used for all analyses. The definition of hypertension was as follows: individuals who reported having diagnosed hypertension or receiving BP-lowering treatment or had an average measured SBP of at least 140 mm Hg, DBP of at least 90 mm Hg, or both [20].

Both body height and weight were measured with the participants in light clothing and without shoes after an overnight fast. Body mass index (BMI) was defined as measured weight in kilograms divided by squared height in meters. There were three BMI categories: underweight defined as a BMI <18.5 kg/m^2^; normal weight, 18.5–23.9 kg/m^2^; overweight and obesity (high BMI), ≥24 kg/m^2^.

Education was categorized as low (receiving only primary education or no education, ≤6 years), middle (finishing secondary school or high school, 9–12 years), and high (graduating from college or university, ≥16 years). Smoking status was classified as never-smokers and ever-smokers which included current smokers (who have been regularly smoking at least one cigarette per day during the previous 6 months) and ex-smokers (who had once smoked but quitted smoking for 6 months or longer).

### 2.3. Statistical Analysis

Summary results were presented as a percentage for categorical variables and mean for continuous variables. Trend analyses were conducted using multiple logistic regression, multiple linear regression, and multinomial logistic regression, where appropriate. All these models were adjusted for age (except age-trend analysis) and sex (except female-trend analysis); time periods were incorporated as a continuous variable, and if the coefficient was statistically significant, we then concluded that it has a linear trend.

We separately investigated the impact of overweight/obesity on hypertension in each time period using multiple logistic regression. Additionally, we also examined the effect of high BMI and time period with combined data. Based on the coefficients of this combined model, we calculated the predicted probability of hypertension by BMI category in each period. All models were adjusted for covariates.

We evaluated whether the associations between overweight/obesity and hypertension among Yi people varied by time periods both on multiplicative and additive scales. The *P*-values of the cross-product interaction term (overweight/obesity X time period) in the multiple logistic models were used to test for the multiplicative interaction [21]. The additive interaction was measured with relative excess risk due to interaction (RERI), using the following equation [21, 22]: RERI=RR_11_ − RR_10_ − RR_01_+1.

We further tested the decomposition of the joint effect, that is, the proportions attributable to overweight/obesity alone, to time period alone, and to their interaction [21, 22].

According to the definition of population attributable fraction (PAF) [23], the PAF in our study estimated the proportion of hypertension that could be attributed to its association with overweight and obesity. It was calculated for each time period by comparing the observed prevalence of hypertension with a counterfactual scenario in which overweight/obese persons experienced the same odds of hypertension as those who had normal weight, adjusting for covariates.

All statistical analyses were performed using SAS software V. 9.4 (SAS Institute) and R3.6.0. Two-sided significance level for analyses was set at *α* = 0.05.

## 3. Results

In total, 11,598 participants (8,749 Yi people) were included in the Yi Migrant Study. After excluding Han people and missing data, there were 8,412 Yi people in the final analysis.  Table 1 shows the prevalence of overweight/obesity (BMI ≥24.0) and the crude prevalence of hypertension within BMI categories. Over the past twenty years, there was an increasing linear trend of overweight/obesity prevalence. Yi migrants had a significantly higher prevalence of high BMI than Yi farmers, and more than half (52.31%) of Yi migrants in 2015 were overweight or obesity. After being adjusted for age and sex, the hypertension prevalence saw a significantly upward linear trend both in the normal weight group (*P* < 0.001) and overweight/obesity group (*P*=0.026).

**Figure 1:**
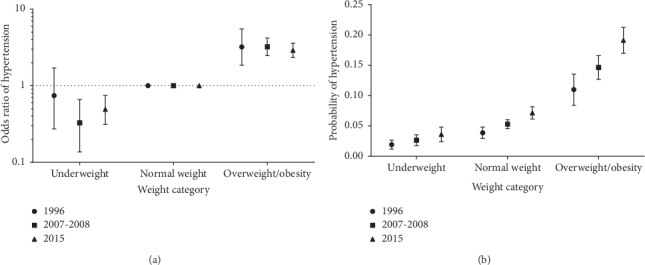
Odds ratio (a) and predicted probability (b) of hypertension according to body mass index (BMI; weight (kg)/height (m)^2^) category (underweight (BMI <18.5), normal weight (BMI 18.5–23.9), overweight/obesity (BMI ≥24.0)) and time period among Yi people, Yi Migrant Study, 1996–2015. Results were adjusted for age, sex, education, and smoking. Error bars represent 95% confidence interval.

Figure 1 displays the adjusted odds ratios and predicted probability of hypertension according to BMI category and time period. When counting the three periods separately, the odds ratios of hypertension for overweight/obesity were 3.21 (95% confidence interval (CI): 1.86, 5.52), 3.23 (95% CI: 2.48, 4.22), and 2.90 (95% CI: 2.34, 3.60) from period 1 to period 3, respectively (Figure 1(a)). Because odds ratios can inflate relative risks with common outcomes, we also calculated prevalence ratios using adjusted predicted probabilities (Figure 1(b)). For overweight/obesity, the adjusted prevalence ratios of hypertension exhibited the same time trend as the odds ratios, and the point estimates were 2.84, 2.77, and 2.67, respectively.

Table 2 provides odds ratios of hypertension using different time periods as reference group among combined participants. In model 1, period 1 was the reference group, and overweight/obesity was a statistically significant risk factor for hypertension, which leads approximately three times higher risk than the normal weight participants. After being adjusted for age, sex, education, smoking, and BMI category, time period was an independent risk factor for hypertension, which indicated that Yi people in period 2 or period 3 had a 1.39 (95% CI: 1.07, 1.83) or 1.92 (95% CI: 1.47, 2.52) times higher risk of hypertension than the same person who lived in period 1. When compared with Yi people in period 2 using model 2, the same person in period 3 had a 1.38 (95% CI: 1.17, 1.63) times higher risk of hypertension, while the risk for those in period 1 was significantly lower (0.72, 95% CI: 0.55, 0.94).

The interactive effects of overweight/obesity and time period on the risk of hypertension are shown in Table 3. For normal weight Yi people, living in period 2 had a 1.46-fold higher risk of hypertension than living in period 1 (95% CI: 1.02, 2.14). The hypertension relative risk of normal weight people in period 3 was 1.75 (95% CI: 1.22, 2.58) times higher than that in period 1 and 1.50 (95% CI: 1.17, 1.91) times higher than that in period 2. For overweight/obesity group in period 2 and period 3, the risk of hypertension increased to 4.90-fold (95% CI: 3.38, 7.24) higher and 5.15-fold (95% CI: 3.62, 7.50) higher than that of the normal weight group in period 1.

Tests for multiplicative interaction were not significant across all groups. Nonetheless, we found significant additive interactions between overweight/obesity and time periods (period 2 and period 3) on the risk of hypertension in Yi people. When using normal weight people in period 1 as the reference group, the relative excess risk due to interaction (RERI) among overweight/obesity participants in period 2 was 1.59 (95% CI: 0.12, 3.05), and in period 3 it was 1.41 (95% CI: 0.30, 2.78). The attributable proportions of joint effect (overweight/obese population in period 2/3) were 40.71% (95% CI: 6.62%, 74.81%) and 33.95% (95% CI: 8.57%, 67.04%) for their interaction in period 2/3, respectively. There was no interaction when comparing period 3 with period 2 (*P*=0.372). All these statistically significant interactions were observed in Yi migrants ([Supplementary-material supplementary-material-1]).

Table 4 shows population attributable fractions (PAFs). We estimated the proportions of hypertension attributable to overweight/obesity in each time period. In agreement with the pattern of increasing high BMI prevalence elucidated in Table 1, the PAF for hypertension showed a considerable increase from period 1 to period 2 and period 3 in Yi people, changing from 27.66% to 33.45% and 33.26%. The trend of an increasing PAF had leveled off in more recent years.

## 4. Discussion

Rapidly increasing overweight and obesity rates, coupled with population ageing, have evoked extensive concern about the consequences of overweight and obesity for health outcomes, such as hypertension. In our study, we found a significant increase in the association between overweight/obesity and hypertension from 1996 to 2015 among Yi people. It suggests that the same weight status is more likely to be hypertensive than in the past, raising the serious concern that overweight/obesity is becoming a more epidemic comorbidity with other cardiovascular disease risk factors.

In line with our findings, Nagai et al. concluded that from 1980 to 2010 the multivariable-adjusted odds ratios for hypertension, comparing overweight and obesity with normal weight, went from 1.94 to 2.82 in men and from 2.37 to 3.48 in women [12]. Data from 1994–2014 National Health and Nutrition Examination Survey (eight survey cycles) also suggested that there was a significant, positive linear trend (*P*=0.006) in the association between overweight/obesity and hypertension among American adults [24]. Moreover, a trend study in Taiwan also revealed that the association between SBP and BMI became stronger for both men and women in 2006 than in 1996 after adjustment for covariates [25].

There are many reasons behind the increasing trend in the relationship between overweight/obesity and hypertension. First, it was suggested that birth cohort could influence our health conditions. A study using data of two British birth cohorts demonstrated that there was a stronger association between BMI trajectory and BP in the later-born cohort [13]. Second, the duration of overweight and obesity was increasing in the population. The Chinese National Survey on Student's Constitution and Health showed that the overweight prevalence continually increased from 1.1% in 1985 to 20.4% in 2014 among Chinese school-aged children (7–18 years) [26]. An earlier age of onset and a longer duration of overweight and obesity would be expected to increase obesity-associated chronic diseases, such as diabetes and hypertension [27, 28]. Third, along with the growth of overweight/obesity prevalence, the mean BMI also experienced a considerable increase, which means that the population distribution of BMI was not increasing uniformly but was increasing in its skew to the right. Consequently, there were more severe obesity patients in the population. A pooled analysis conducted by NCR Risk Factor Collaboration predicted that if present trends continue, by 2025 severe obesity will surpass 9% in women and 6% in men and will be larger than the projected prevalence of underweight in women [29]. The dose response between BMI and risk of hypertension [30] indicates a stronger effect of severe obesity on hypertension. Furthermore, cumulative exposure to overweight and obesity would cause related disability [31], which could in turn interact with high BMI on the risk of hypertension [32].

We also found that the increasing association between overweight/obesity and hypertension has leveled off in more recent years. A Germany national survey found that the association between BMI and SBP tended to level out between 1998 and 2011, which could be partly explained by the improvements in the diagnosis and treatment of high blood pressure [33]. Prior work suggested that physicians may be more aggressive in managing risk factors and preventive care for obese patients relative to normal weight patients [34]. Additionally, patients who are obese have a higher frequency of clinical visits and may seek medical care earlier in the course of the disease [35].

Our work showed that normal weight Yi people saw a continuous increase in the risk of hypertension during the past twenty years. This could be due to changes among other uninvolved risk factors of hypertension. It is reported that dietary acid load was significantly and positively associated with hypertension among normal weight people [36]. China Health and Nutrition Survey indicated that there was a dramatic change in dietary pattern in the past two decades; nowadays people are more likely to consume processed food with refined carbohydrates, added salt, and sweetener, while the average intake of cereal, fresh fruits, and vegetables has decreased [37]. Another potential reason for the increase in hypertension risk among normal weight Yi people is the reduced physical activity. A systematic review supported there was an inverse association between physical activity and incident hypertension [38]. With the rapid process of urbanization and modernization in China, most Yi people reduced the strength of physical activity compared with what they did before.

In addition to overweight and obesity, there are many other environmental risk factors which may contribute to the increasing trends of hypertension prevalence in Yi people. Sodium intake is positively associated with BP [39]. In a community-based survey in southwestern China [40], the authors found that Yi migrants displayed a higher BP level than Yi farmers, which could be explained by the higher sodium-to-potassium ratio among Yi migrants. Furthermore, the detrimental effect of alcohol consumption on BP has been extensively studied, and estimates of the contribution of alcohol intake on hypertension risk vary according to the level of consumption [41]. Yi people were characterized by heavy drinking and high-salt diet; therefore, these dietary factors could partially explain the prevalence of hypertension in recent years. Recently, air pollution is becoming an important risk factor of hypertension in China. An epidemiological study demonstrated that the odds ratio for hypertension increased by 1.12 (95% CI, 1.08–1.16) per 19 *μ*g/m^3^ increase in PM_10_ in China [42]. Urban areas are normally more polluted than rural areas; this may explain why Yi migrants were more likely to be hypertensive than Yi farmers.

There were limitations to this study. First, this is a cross-sectional study, and little is known about the magnitude of a potential causal effect of hypertension on overweight and obesity. The bidirectional relationship would strengthen their association. Our goal was to examine how the overall association has changed over time; therefore recognizing these changes with interaction analysis could also include the inverse effect from hypertension. Second, the tools for measuring BP were not consistent among the three time periods. However, the automatic devices had been calibrated with the Mercury sphygmomanometer before measurement, and some studies have validated the accuracy and validation of Omron HEM-907 [43]. At last, we did not include Han people in each period. Therefore, the comparison between the two ethnic groups could not be implemented.

To our knowledge, this is the first study to investigate the secular trends of the impact of overweight and obesity on hypertension among Yi people. We also compared the differences between Yi migrants and Yi farmers in this issue. Moreover, these three successive Yi migrant studies were almost completed by one stable team using the same protocol, with only a few students renewed, and this high consistency guarantees the comparability among different time periods.

In summary, there were significant increases in the association between overweight/obesity and hypertension from 1996 to 2015 among Yi people; this trend has leveled off in more recent years. The comorbidity of overweight/obesity and hypertension would greatly aggravate the risk of cardiovascular disease. The continuously increased risk of hypertension in normal weight Yi people should be taken into adequate account. Further studies should aim to identify reasons behind these increasing trends and add some mediation analyses to assist us to elucidate pathways among comorbidity diseases.

## Figures and Tables

**Table 1:** Characteristics of participants by body mass index category and time period, Yi Migrant Study, 1996–2015.

Variable	BMI^a^ category and time period^b^											
	Underweight (BMI <18.5)				Normal weight (BMI 18.5–23.9)				Overweight/obesity (BMI ≥24.0)			
Yi people	P1 *n* = 179	P2 *n* = 313	P3 *n* = 314	*P* for trend^c^	P1 *n* = 1,084	P2 *n* = 2,511	P3 *n* = 1,696	*P* for trend^c^	P1 *n* = 218	P2 *n* = 840	P3 *n* = 1,257	*P* for trend^c^
Prevalence (%)	12.09	8.54	9.61	0.356	73.19	68.53	51.91	<0.001	14.72	22.93	38.48	<0.001
BMI (kg/m^2^)	17.54	17.59	17.27	0.117	20.94	21.22	21.32	<0.001	26.09	26.60	27.04	<0.001
Age, years	37.21	41.31	50.32	<0.001	33.82	38.52	45.23	<0.001	40.69	41.35	47.16	<0.001
Female (%)	44.13	51.76	66.24	<0.001	37.92	49.74	66.80	<0.001	39.91	46.43	66.43	<0.001
Hypertension (%)	3.35	2.24	9.24	0.022	3.69	5.69	11.56	<0.001	15.14	21.67	26.73	0.026

Yi farmers	*n* = 122	*n* = 213	*n* = 242		*n* = 649	*n* = 1,806	*n* = 1,107		*n* = 26	*n* = 283	*n* = 532	
Prevalence (%)	15.31	9.25	12.86	0.371	81.43	78.45	58.85	<0.001	3.26	12.29	28.28	<0.001
BMI (kg/m^2^)	17.45	17.55	17.28	0.549	20.64	21.11	21.11	<0.001	26.21	25.95	26.83	<0.001
Age, years	38.84	44.25	50.59	<0.001	32.15	38.90	44.36	<0.001	33.81	38.55	44.35	<0.001
Female (%)	38.52	51.17	66.94	<0.001	34.67	50.94	65.31	<0.001	50.00	66.78	66.54	0.307
Hypertension (%)	2.46	2.35	8.68	0.016	3.08	4.82	9.76	<0.001	7.69	12.72	20.86	0.076

Yi migrants	*n* = 57	*n* = 100	*n* = 72		*n* = 435	*n* = 705	*n* = 589		*n* = 192	*n* = 557	*n* = 725	
Prevalence (%)	8.33	7.34	5.19	0.734	63.60	51.76	42.50	<0.001	28.07	40.89	52.31	<0.001
BMI (kg/m^2^)	17.73	17.66	17.22	0.0333	21.4	21.5	21.73	0.047	26.07	26.92	27.20	<0.001
Age, years	33.74	35.06	49.44	<0.001	36.31	37.53	46.88	<0.001	41.62	42.77	49.22	<0.001
Female (%)	56.14	53.00	63.89	0.119	42.76	46.67	69.61	<0.001	38.54	36.09	66.34	<0.001
Hypertension (%)	5.26	2.00	11.11	0.855	4.60	7.94	14.94	0.013	16.15	26.21	31.03	0.014

Data are presented as mean or percentage, where appropriate. BMI: body mass index; P1: period 1; P2: period 2; P3: period 3. a: weight (kg)/height (m)^2^. b: period 1, 1996; period 2, 2007–2008; period 3, 2015. c: *P*-values for trend were adjusted age (except age-trend analysis) and sex (except female-trend analysis) using multiple linear regression, multiple logistic regression, and multinomial logistic regression, where appropriate.

**Table 2:** Relative odds of hypertension in Yi people, Yi Migrant Study, 1996–2015^a^.

Variable	Model 1						Model 2^b^					
	Total		Yi farmers		Yi migrants		Total		Yi farmers		Yi migrants	
	OR	95% CI	OR	95% CI	OR	95% CI	OR	95% CI	OR	95% CI	OR	95% CI
BMI^c^ category												
Underweight (BMI <18.5)	0.51	0.34, 0.68	0.50	0.32, 0.75	0.52	0.27, 0.93	0.51	0.34, 0.68	0.50	0.32, 0.75	0.52	0.27, 0.93
Normal weight (BMI 18.5–23.9)	1.00	Referent	1.00	Referent	1.00	Referent	1.00	Referent	1.00	Referent	1.00	Referent
Overweight/obesity (BMI ≥24.0)	2.89	2.62, 3.59	2.86	2.22, 3.67	2.93	2.37, 3.63	2.89	2.62, 3.59	2.86	2.22, 3.67	2.93	2.37, 3.63
Time period												
Period 1 (1996)	1.00	Referent	1.00	Referent	1.00	Referent	0.72	0.55, 0.94	0.77	0.48, 1.19	0.62	0.44, 0.87
Period 2 (2007–2008)	1.39	1.07, 1.83	1.30	0.85, 2.08	1.61	1.15, 2.29	1.00	Referent	1.00	Referent	1.00	Referent
Period 3 (2015)	1.92	1.47, 2.52	2.18	1.41, 3.49	1.82	1.30, 2.59	1.38	1.17, 1.63	1.67	1.31, 2.15	1.13	0.89, 1.43

BMI: body mass index; OR: odds ratio; CI: confidence interval. a: all models were adjusted for age, sex, education, and smoking. b: model 2 rearranged period 2 as reference group and otherwise had the same parameters as model 1. c: weight (kg)/height (m)^2^.

**Table 3:** Interactions between overweight/obesity and time period among Yi people in association with risk of hypertension, Yi Migrant Study, 1996–2015^a^.

	P2 vs. P1		P3 vs. P1		P3 vs. P2	
	OR	95% CI	OR	95% CI	OR	95% CI
Main effects						
Overweight/obesity (BMI^b^ ≥24)	2.85	1.70, 4.74	2.99	1.78, 4.99	3.56	2.75, 4.60
Time period	1.46	1.02, 2.14	1.75	1.22, 2.58	1.50	1.17, 1.91
Joint effect	4.90	3.38, 7.24	5.15	3.62, 7.50	4.21	3.35, 5.29
Relative excess risk due to interaction (RERI)	1.59	0.12, 3.05	1.41	0.30, 2.78	0.15	−0.76, 1.07
*P* value	0.017		0.027		0.372	
Attributable proportion, %						
Overweight/obesity (BMI ≥24)	47.41	16.65, 78.17	47.91	17.71, 78.10	79.78	54.50, 105.06
Time period	11.88	1.56, 22.19	18.14	8.00, 28.28	15.46	6.14, 24.78
Additive interaction	40.71	6.62, 74.81	33.95	8.57, 67.04	4.76	−23.37, 32.88
Multiplicative interaction	1.18	0.67, 2.07	0.98	0.57, 1.72	0.79	0.57, 1.10

P1: period 1; P2: period 2; P3: period 3; CI: confidence interval; OR: odds ratio; BMI: body mass index. a: all models were adjusted for age, sex, education, and smoking. b: weight (kg)/height (m)^2^.

**Table 4:** Population attributable fraction of overweight/obesity for hypertension in Yi people, Yi Migrant Study, 1996–2015^a^.

Time period	Yi people		Yi farmers		Yi migrants	
	PAF, %	95% CI	PAF, %	95% CI	PAF, %	95% CI
Period 1 (1996)	27.66	13.41, 41.90	4.73	-6.52, 15.98	35.79	15.26, 56.32
Period 2 (2007–2008)	33.45	25.95, 41.03	20.10	11.45, 28.75	43.43	31.99, 54.86
Period 3 (2015)	33.26	26.79, 39.73	30.13	21.62, 38.65	35.44	25.74, 45.13

PAF: population attributable fraction; CI: confidence interval. a: all models were adjusted for age, sex, education, and smoking.

## Data Availability

The data used to support the findings of this study are available from the corresponding author upon reasonable request.

## Supplementary Materials

Supplementary Materials Table S1: interactions between overweight/obesity and time period in association with risk of hypertension by migration status, Yi Migrant Study, 1996–2015.
