# Supplementary material for: Secular Trends of the Impact of Overweight and Obesity on Hypertension in Yi People: Yi Migrant Study, 1996–2015
Source: Int J Hypertens. 2020 Mar 29;2020:5368357. doi: 10.1155/2020/5368357 (PMC7146090; doi:10.1155/2020/5368357)
Supplement: Supplementary Materials — Table S1: interactions between overweight/obesity and time period in association with risk of hypertension by migration status, Yi Migrant Study, 1996–2015. [file 5368357.f1.docx]

| Table S1. Interactions Between Overweight/Obesity and Time Period in Association with Risk of Hypertension by Migration Status, Yi Migrant Study, 1996-2015^a^ | | | | | | | | | | | | |
| --- | --- | --- | --- | --- | --- | --- | --- | --- | --- | --- | --- | --- |
|  | P2 VS P1 | | | | P3 VS P1 | | | | P3 VS P2 | | | |
|  | Yi Farmers | | Yi Migrants | | Yi Farmers | | Yi Migrants | | Yi Farmers | | Yi Migrants | |
|  | OR | 95% CI | OR | 95% CI | OR | 95% CI | OR | 95% CI | OR | 95% CI | OR | 95% CI |
| Main Effects |  |  |  |  |  |  |  |  |  |  |  |  |
| High BMI^b^ (BMI ≥24) | 2.17 | 0.32, 8.80 | 3.05 | 1.67, 5.69 | 2.61 | 0.39, 10.48 | 2.94 | 1.60, 5.50 | 3.34 | 2.12, 5.19 | 3.23 | 2.30, 4.61 |
| Time Period | 1.42 | 0.87, 2.43 | 1.69 | 1.04, 2.98 | 1.82 | 1.10, 3.15 | 1.87 | 1.10, 3.30 | 1.65 | 1.21, 2.26 | 1.27 | 0.86, 1.88 |
| Joint effect | 4.17 | 2.28, 7.83 | 5.46 | 3.35, 9.34 | 5.60 | 3.36, 9.74 | 5.10 | 3.12, 8.74 | 4.81 | 3.48, 6.66 | 3.36 | 2.40, 4.78 |
| Relative excess risk due to interaction  (RERI) | 1.58 | -2.11, 5.27 | 1.72 | 0.16, 3.51 | 2.17 | -2.01, 6.36 | 1.29 | 0.32, 2.91 | 0.82 | -0.87, 2.51 | -0.14 | -1.14, 0.86 |
| *P*-value | 0.201 | | 0.029 | | 0.155 | | 0.031 | | 0.171 | | 0.607 | |
| Attributable Proportion, % |  |  |  |  |  |  |  |  |  |  |  |  |
| High BMI (BMI ≥24) | 36.93 | -68.59, 142.45 | 45.87 | 15.29, 76.46 | 34.95 | -51.15, 121.04 | 47.29 | 15.15, 79.42 | 61.47 | 23.84, 99.10 | 94.57 | 56.07, 133.08 |
| Time Period | 13.37 | -4.03, 30.77 | 15.54 | 1.16, 29.93 | 17.84 | 4.68, 30.99 | 21.16 | 5.65, 36.68 | 17.02 | 5.48, 28.56 | 11.28 | -6.45, 29.02 |
| Additive interaction | 49.70 | -59.32, 158.73 | 38.58 | 2.28, 74.88 | 47.21 | -40.84, 135.28 | 31.55 | 6.25, 69.35 | 21.51 | -19.93, 62.95 | -5.85 | -48.49, 36.77 |
| Multiplicative Interaction | 1.35 | 0.31, 9.55 | 1.06 | 0.52, 2.13 | 1.18 | 0.28, 8.14 | 0.93 | 0.46, 1.84 | 0.87 | 0.51, 1.50 | 0.82 | 0.52, 1.30 |
| Abbreviations: P1, period 1; P2, period 2; P3, period 3; CI, confidence interval; OR, odds ratio; BMI, body mass index  a All models adjusted for age, sex, education, and smoking.  b Weight (kg)/height (m)^2^ | | | | | | | | | | | | |
